# An Inactivated Influenza Virus Vaccine Approach to Targeting the Conserved Hemagglutinin Stalk and M2e Domains

**DOI:** 10.3390/vaccines7030117

**Published:** 2019-09-18

**Authors:** Weina Sun, Allen Zheng, Robert Miller, Florian Krammer, Peter Palese

**Affiliations:** 1 Department of Microbiology, Icahn School of Medicine at Mount Sinai, New York, NY 10029, USA; allen.zheng@icahn.mssm.edu (A.Z.); robert.miller@mssm.edu (R.M.); florian.krammer@mssm.edu (F.K.); 2 Department of Medicine, Icahn School of Medicine at Mount Sinai, New York, NY 10029, USA

**Keywords:** cross-protection, ADCC, conserved epitopes, universal influenza virus vaccine, enhanced immunogenicity, immunodominance

## Abstract

Universal influenza virus vaccine candidates that focus on the conserved hemagglutinin (HA) stalk domain and the extracellular domain of the matrix protein 2 (M2e) have been developed to increase the breadth of protection against multiple strains. In this study, we report a novel inactivated influenza virus vaccine approach that combines these two strategies. We inserted a human consensus M2e epitope into the immunodominant antigenic site (Ca2 site) of three different chimeric HAs (cHAs). Sequential immunization with inactivated viruses containing these modified cHAs substantially enhanced M2e antibody responses while simultaneously boosting stalk antibody responses. The combination of additional M2e antibodies with HA stalk antibodies resulted in superior antibody-mediated protection in mice against challenge viruses expressing homologous or heterosubtypic hemagglutinin and neuraminidase compared to vaccination strategies that targeted the HA stalk or M2e epitopes in isolation.

## 1. Introduction

The matrix protein 2 (M2) of influenza A viruses is a tetrameric type III integral transmembrane protein comprised of an N-terminal ectodomain (M2e, aa 1–24), a transmembrane domain (aa 25–44) and a C-terminal cytoplasmic tail (aa 45–97) [1]. It is expressed from the spliced mRNA of the M segment [2,3], and it has been reported to play important roles in virus entry and egression [4]. Specifically, after the virus is endocytosed, the ion channel activity of the M2 proteins allows for the acidification of the virion interior within the endosomes, resulting in disassembly of the viral particles and release of the viral genomic segments. On the other hand, at the end of the viral life cycle, the amphipathic helices in the cytoplasmic tail of M2 can initiate membrane scission independent of the host’s machinery to facilitate budding [5,6]. The M2 protein is highly conserved across all influenza A viruses [4]. In contrast to the other two surface glycoproteins of the virion—hemagglutinin (HA) and neuraminidase (NA)—the immunogenicity of M2 is poor, resulting in weak or not-detectable M2e-specific antibody responses after vaccination with an inactivated influenza virus vaccine or even live-virus infections in animal models [7,8,9] or humans [10,11]. This is likely due to its small size and low copy number in the virions [12]. Despite this, M2e-specific monoclonal antibodies have been reported to restrict virus growth in vitro and in vivo. Several of these are known to be cross-reactive, and confer broad protection against heterosubtypic influenza virus challenge in animal models [13,14,15,16,17,18,19,20,21,22,23,24,25].

Many universal vaccination strategies have attempted to increase the immunogenicity of the M2 protein because of this protein’s similarity across all influenza A viruses [5,26,27,28,29,30,31,32,33]. Well-characterized vaccine candidates include virus-like particles (VLPs) expressing the M2 protein, such as M2eHBc VLPs [33,34,35,36,37], M2e5 x (tandem repeats) VLPs [38,39,40,41,42,43,44] and 4.M2e-tFliC/M1 VLPs containing flagellin as toll-like receptor (TLR) ligand [45]; soluble, recombinant M2 protein alone or in combination with other influenza viral antigens, such as soluble M2e with tGCN4 tetrameric domains (M2e-tGCN4) [46] and flagellin-fused M2e plus HA2 proteins [47,48]; or recombinant live viral vectors expressing M2e, such as M2e-expressing adenovirus [49,50,51], M2e-expressing Modified Vaccinia Virus Ankara (MVA) [52] and a T7-bacteriaphage displaying the M2e [53]. Unfortunately, none of these strategies are compatible with currently accepted platforms of live-attenuated or inactivated virus vaccines.

To increase the immune response against M2e for broader protection in the context of inactivated virus vaccination, we generated recombinant influenza viruses in the A/Puerto Rico/08/1934 (PR8) backbone that display a consensus human M2 epitope within one of the major antigenic sites of the H1 hemagglutinin. By immunizing mice with this modified inactivated virus (PR8 Ca2 M2), the M2e epitope can elicit strong non-neutralizing M2e-specific antibody responses that are protective against a virus expressing the heterosubtypic HA and NA. Moreover, we decided to combine this approach with our previously developed chimeric HA (cHA) approach—a universal vaccination strategy that boosts anti-HA stalk antibody responses through sequential vaccination with viruses expressing HAs with the same stalk but different heads [54,55,56,57,58,59,60,61,62]. The same M2e epitope was inserted into the putative Ca2 antigenic sites of cHAs containing identical stalks but different exotic head domains. We observed that sequential immunization with modified inactivated recombinant cHA Ca2 M2 viruses significantly increased the M2e-specific antibody level while also boosting stalk antibody levels. As expected, the cHA Ca2 M2 strategy showed an enhanced M2e antibody titer and protected mice from a challenge virus with heterosubtypic HA and NA more effectively than the repeated immunizations with PR8 Ca2 M2 virus (expressing the M2e epitope) alone. The combination of M2e antibodies and stalk antibodies generated by the cHA Ca2 M2 viruses also protected mice against a homologous virus challenge significantly better than the cHA approach alone. 

## 2. Materials and Methods

### 2.1. Ethics Statement

All animal studies were performed in accordance with protocol (#06-0218-00001-02) approved by the Institutional Animal Care and Use Committee (IACUC) at the Icahn School of Medicine at Mount Sinai. All animals were housed in a temperature-controlled biosafety level 2 (BSL-2) animal facility at the Annenberg building. All efforts were made to minimize animal suffering.

### 2.2. Cells

Human embryonic kidney 293T (HEK 293T) cells were maintained in Dulbecco’s Modified Eagle’s medium (DMEM; Gibco) containing 10% (vol/vol) fetal bovine serum (FBS) and 100 unit/mL of penicillin/streptomycin (PS; Gibco) at 37 °C with 5% CO_2_. Madin–Darby canine kidney (MDCK) cells were grown in Minimum Essential Medium (MEM; Gibco) supplemented with 10% (vol/vol) FBS, 2 mM of L-glutamine (Gibco), 0.15% (w/vol) of sodium bicarbonate (Corning), 20 mM of 2-[4-(2-hydroxyethyl)piperazin-1-yl] ethanesulfonic acid (HEPES, Gibco), and 100 unit/mL of PS at 37 °C with 5% CO_2_. To generate MDCK cells stably expressing the PR8 M2 protein, a 6-well plate with MDCK cells was transfected with 5 µg of pCAGGS PR8 M2 plasmid and 1 µg of pTK-Hyg plasmid per well using TransIT LT1 (Mirus Bio, Madison, WI, USA). One well of transfected cells were split into eight 20 cm dishes and supplemented with 100 µg/mL of hygromycin B (Thermo Fisher Scientific, Waltham, MA, USA) for selection. One to two weeks later, colonies were picked and cultured into a 96-well plate for immunostaining with the E10 mouse monoclonal antibody (anti-M2, Center for Therapeutic Antibody Development at Icahn School of Medicine at Mount Sinai). Positive clones were expanded into T175 flasks supplemented with 100 µg/mL of hygromycin B and 5 µM of amantadine hydrochloride (Sigma). 

### 2.3. Plasmids

The M2 epitope (5’-AGTCTTCTAACCGAGGTCGAAACGCCTATCAGAAACGAATGGGGG-3’) was introduced into the Ca2 or putative Ca2 antigenic sites of the HAs through PCR to generate plasmids expressing the PR8 Ca2 M2, cH5/1 Ca2 M2, cH8/1 Ca2 M2 and the cH11/1 Ca2 M2 hemagglutinin genes. For example, to generate PR8 Ca2 M2 HA, the nucleotide sequence of the M2e insertion was split into two primers—the M2e insert reverse primer (5’-GATAGGCGTTTCGACCTCGGTTAGAAGACTCTCATGGGAGCATGCTGCCG-3’) and the M2e insert forward primer (5’-GTCGAAACGCCTATCAGAAACGAATGGGGGGGGAAAAGCAGTTTTTACAG-3’), which have 15 nucleotides of overlap for In-Fusion cloning (Takara Bio). Two segments were amplified for each HA gene, the 5’ segment using the PR8 HA NCR forward (5’-CCGAAGTTGGGGGGGAGCAAAAGCAGGGGAAAATA-3’) and M2e insert reverse primers, and the 3’ segment using the M2e insert forward and the PR8 HA NCR reverse (5’-GGCCGCCGGGTTATTAGTAGAAACAAGGGTGTTTTTC-3’) primers. Both PR8 HA NCR forward and PR8 HA NCR reverse primers contain 15 nucleotides of overlap with the end sequences of the linearized vector, which was generated by SapI restriction enzyme (New England Biolabs, Inc., Ipswich, MA, USA) digestion of the pDZ ambisense plasmid [63]. The two modified HA segments were subsequently cloned into the pDZ vector through In-Fusion cloning (Takara Bio, Kusatsu, Shiga Prefecture, Japan). The recombination products were transformed into *Escherichia coli* DH5α competent cells (Thermo Fisher Scientific) and plasmids were purified using QIAprep Spin Miniprep kit (Qiagen). All the other cHA Ca2 M2 plasmids were generated using the same approach. The pCAGGS PR8 M2 plasmid was constructed by amplifying the M2 opening reading frame (ORF) sequence from the PR8 M segment through PCR and subcloning the M2 ORF into a mammalian expression vector-pCAGGS. Sequences of HA or M2 gene were confirmed by Sanger sequencing (Macrogen). The pRS PR8 7 segment plasmid used to rescue recombinant influenza viruses has been described previously [64]. 

### 2.4. Rescue of the Recombinant Influenza Viruses

Each well of poly-D lysine (Sigma) coated 6-well plates of HEK 293T cells was transfected with 2.8 µg of pRS PR8 7 segment, 0.7 µg of modified pDZ HA and 0.5 µg of pCAGGS PR8 HA helper plasmid using TransIT LT1 transfection reagent (Mirus Bio). Transfected cells were incubated at 37 °C. Forty-eight hours post-transfection, supernatants together with scraped cells were collected and briefly homogenized through several syringe strokes. Two-hundred microliters of cells and supernatant mixture were injected into the allantoic cavity of 8-day old embryonated chicken eggs (Charles River). Injected eggs were incubated at 33 °C for 3 days and then cooled at 4 °C overnight. Allantoic fluids were subsequently collected and clarified by low speed centrifugation. An HA assay was performed using 0.5% turkey red blood cells to examine the presence of rescued virus from the clarified allantoic fluids. HA positive allantoic fluid samples were used to plaque-purify virus on MDCK cells. Plaques grown on MDCK cells were picked and re-suspended in PBS and further amplified again in 10-day old embryonated chicken eggs. RNA was extracted from allantoic fluids containing the plaque-purified virus using QIAamp Viral RNA Mini Kit (Qiagen). One-step RT-PCR was performed to amplify DNA of the HA segment using the SuperScript™ III One-Step RT-PCR System with Platinum™ Taq DNA Polymerase (Thermo Fisher Scientific) and HA specific primers. DNA was gel-purified and sequenced by Sanger sequencing (Genewiz). All the viruses were rescued in the PR8 backbone (7 genomic segments except HA are from PR8). All the cHAs had the stalk domain from A/California/04/2009 (Cal09) pdm H1N1 hemagglutinin. The head domains of cHAs were from A/Vietnam/1203/2004 H5N1-PR8-IBCDC-RG/GLP hemagglutinin (cH5/1), A/mallard/Sweden/24/2002 H8N4 hemagglutinin (cH8/1) or A/shoveler/Netherlands/18/1999 H11N9 hemagglutinin (cH11/1). The reason that H5, H8 and H11 head domains are chosen for sequential immunization is that humans are normally naïve to these exotic avian hemagglutinins and that they are very different from each other, which is necessary to redirect the immune system to the conserved epitopes. A virus with full length wild type Cal09 HA was also rescued in the PR8 backbone (WT Cal09 HA PR8).

### 2.5. Inactivation and Purification of Influenza Viruses

Influenza viruses were grown in 10-day old embryonated chicken eggs at 37 °C for two days, and were then cooled at 4 °C overnight. Allantoic fluids were collected and clarified by low speed centrifugation. Viruses in the clarified allantoic fluids were inactivated with 0.03% methanol-free formaldehyde for 48 h at 4 °C with rocking. Viruses were then pelleted through a 30% sucrose cushion in NTE buffer (100 mM NaCl, 10 mM Tris-HCl, 1 mM EDTA, pH 7.4) by centrifugation in a Beckman L7-65 ultracentrifuge at 25,000 rpm for 2 h at 4 °C using a Beckman SW28 rotor (Beckman Coulter, Brea, CA, USA). Pellets were collected in PBS (pH 7.4), and protein content was quantified using the bicinchoninic acid (BCA) assay (Thermo Fisher Scientific).

### 2.6. Mice Immunizations

To mimic inactivated influenza virus vaccination in humans, six to eight-week-old female BALB/c mice were immunized with 10 µg of inactivated and purified virus in 50 µL PBS with 50 µL of AddaVax (Invivogen, San Diego, CA, USA), which is a squalene-based oil-in-water emulsion equivalent to a licensed influenza virus vaccine adjuvant in Europe—MF59 [65]. The virus and adjuvant mixtures were administered intramuscularly with a total volume of 100 µL (50 µL per leg). For a proof of principle immunization study, three groups of mice were included (*n* = 5)—the PR8 WT group; the PR8 Ca2 M2 group; and a naïve group that did not receive any immunogen, nor adjuvant. Mice were boosted once in four-week intervals with the same immunogen. For the cHA Ca2 M2 study, five groups of mice were included (*n* = 8). Mice were boosted twice in four week-intervals. The WT Cal09 HA group received the WT Cal09 HA PR8 virus three times; the PR8 Ca2 M2 group received the PR8 Ca2 M2 virus three times; the cHA group was primed with cH5/1 virus and then boosted by cH8/1 virus and then cH11/1 virus; the cHA Ca2 M2 group was primed with cH5/1 Ca2 M2 virus, boosted by cH8/1 Ca2 M2 virus, and then by cH11/1 Ca2 M2 virus. Mice were bled before each boost. Two weeks after the last boost, mice were terminally bled by cardiac puncture. Sera were isolated by low speed centrifugation and stored at −80 °C before use. Blood samples before the first boost, second boost and terminal bleed were designated as blood samples after 1st, 2nd and 3rd immunization.

### 2.7. Passive Transfer and Viral Challenge Study

Equal amounts of sera from each mouse within each group were pooled. Pooled sera were transferred intraperitoneally (200 µL per mouse for X-31 (a reassortant virus carrying the HA and NA genes of A/Hong Kong/1/1968 H3N2 in the PR8 backbone) challenge; 200 µL per mouse for A/Hong Kong/1/68-2-MA21-2 H3N2 challenge; 100 µL per mouse for A/Netherland/602/2009 pH1N1 challenge;) into groups of naïve 6-to-8-week old female BALB/c mice (*n* = 5). Two-hours post-transfer, animals were anesthetized and challenged with 5 x median lethal dose (LD_50_) of X-31, A/Hong Kong/1/68-2-MA21-2 (H3N2) (BEI Resources) or A/Netherland/602/2009 (pdm H1N1). The M2e sequences of these viruses are shown in Table 1. For the X-31 challenge, three groups of mice receiving the naïve sera, the WT PR8 sera and the PR8 Ca2 M2 sera were included (*n* = 5). For A/Netherland/602/2009 and A/Hong Kong/1/68-2-MA21-2 (H3N2) challenge studies, five groups of mice receiving the following sera were included (*n* = 5): the WT Cal09 HA sera, the PR8 Ca2 M2 sera, the cHA sera, the cHA Ca2 M2 sera and the naïve sera (negative control). Weight loss and survival were monitored to determine serum antibody-mediated protection. Mice were scored dead and euthanized when they lost more than 25% of their body weight. Weight-loss in mice was graphed using GraphPad Prism 7.0. 

### 2.8. Micro-Neutralization Assay

MDCK cells were plated at a concentration of 1.8 × 10^4^ cells per well in a 96-well plate and left to grow overnight at 37 °C, 5% CO2 until they reached 80–90% confluency. The sera were treated with receptor destroying enzyme (RDE) as previously described [66,67], which resulted in 1:10 dilution of the original sera. The RDE treated sera was two-fold serially diluted in 1x Minimal Essential Medium (MEM; 10% 10X MEM, 1.6% of a 7.5% sodium bicarbonate stock solution (pH 7.5), 1% of penicillin/streptomycin antibiotic cocktail (Pen/Strep, Gibco), 1% 200 mM L-glutamine, 1% of a 1MHEPES stock solution and 0.6% of a 35% bovine serum albumin (BSA) stock solution) containing 1 µg/mL L-1-tosylamide-2-phenylethyl chloromethyl ketone (TPCK)-treated trypsin. One-hundred times the median tissue culture infectious dose (TCID50) of A/Hong Kong/4801/2014 (H3N2) NYMC X-263B virus was incubated with half the volume of the serial dilutions at a 1:1 volume ratio for one hour at room temperature with shaking. MDCK cells were washed once with sterile PBS and then the virus-sera mixture was added onto the cells and incubated for one hour at 37 °C. The remaining half of the serial sera dilutions were supplemented with a 1:1 addition of 1 x MEM with 1 µg/mL TPCK-treated trypsin. After 1 h of incubation, the virus-serum mixture was removed and cells were washed again with PBS. The serial serum dilutions were added to the MDCK cells and incubated for 2 days at 37 °C. An HA assay was performed (using 0.5% turkey red blood cells) as the readout for the microneutralization assay to sensitively measure viral replication in the absence of the cytopathic effect (CPE). The microneutralization titer was determined as the last well in which hemagglutination inhibition occurred and graphed using GraphPad Prism 7.0. The M2e sequence of the A/Hong Kong/4801/2014 (H3N2) NYMC X-263B virus is shown in Table 1.

### 2.9. Enzyme-Linked Immunosorbent Assay (ELISA)

Immulon 4 HBX 96-well ELISA plates (Thermo Fisher Scientific) were coated with 2 µg/mL of recombinant proteins (50 µL per well) in coating buffer (SeraCare Life Sciences Inc., Milford, MA, USA) overnight at 4 °C. The next day, all plates were washed three times with 225 µL PBS containing 0.1% (*v*/*v*) Tween-20 (PBST), and 220 µL blocking solution (3% goat serum, 0.5% non-fat dried milk powder and 96.5% PBST) was added to each well and they were incubated for 1 h at room temperature (RT). Mouse sera were 3-fold serially diluted in blocking solution starting at 1:20 for cH6/1_Cal09_ ELISA, 1:100 for PR8 HA and Cal09 HA ELISAs, followed by a 2 h incubation at RT. ELISA plates were washed 3 times with PBST and 50 µL of anti-mouse IgG-horseradish peroxidase (HRP) conjugated antibody (GE Healthcare, Chicago, IL, USA) was added at a dilution of 1:3000 in blocking solution. Then, plates were again incubated for 1 h at RT. Plates were washed 4 times with PBST, and 100 µL of *o*-phenylenediamine dihydrochloride (SigmaFast OPD, Sigma) substrate was added per well. After 10 min, 50 µL of 3 M HCl was added to each well to stop the reaction, and the optical density (OD) was measured at 492 nm on a Synergy 4 plate reader (BioTek, Winooski, VT, USA). The area under the curve (AUC) readout was used to evaluate the total IgG response. An average of the OD values for blank wells, plus three standard deviations, was used to set a cutoff to calculate AUC. A cutoff value was established for each plate. The AUCs of serum IgG responses was graphed using GraphPad Prism 7.0. To perform cell-based ELISAs to measure M2e antibody responses, 293T cells or PR8 M2-expressing MDCK cells were seeded into 96-well tissue culture plates (poly-D lysine coated for 293T cells) at 50,000 cells per well. 293T cells were transiently transfected with pCAGGS PR8 M2 (200 ng/well) the next day and fixed with 4% methanol-free formaldehyde for 30 min at room temperature 24 h post-transfection. MDCK cells were fixed 24 h after seeding with 4% methanol-free formaldehyde for 30 min at room temperature. Cells were then washed 3 times with PBS. The rest of the ELISAs were performed as described above, starting with a 1:20 sera dilution. The cell-based ELISAs were performed with gentle pipetting and washing steps to keep the cell monolayers intact. 

### 2.10. Antibody Dependent Cell-Mediated Cytotoxicity (ADCC) Reporter Assay

The ADCC Reporter Bioassay Kit (Promega Life Sciences) was used to measure the induction of ADCC by serum antibodies. The 293T cells were seeded on poly-D lysine coated, flat bottom white 96-well tissue culture plates (Costar) at 50,000 cells per well the day before transfection. Twenty-four hours later, cells were transfected with pCAGGS PR8 M2 (200 ng/well). The next day, transfected cells were washed with 100 µL of PBS and supplemented with 25 µL of Roswell Park Memorial Institute (RPMI) medium (Thermo Fisher Scientific). Pooled mouse sera were diluted 1:3 (from a starting dilution of 1:30) in RPMI medium and added (25 µL per well) to the transfected cells in triplicates. ADCC mouse effector cells (Promega Life Sciences) were added at a concentration of 75,000 cells per well and incubated for 6 h at 37 °C. At the end of the incubation, 75 µL of Bio-Glo luciferase assay substrate (Promega Life Sciences) was added to each well and incubated at RT for 5 min. Luminescence was read using a Synergy 4 microplate reader (BioTek) and Gen5 2.09 software. Fold induction over baseline was calculated and graphed using Prism 7.0.

### 2.11. Statistics

Statistical analysis was performed using GraphPad Prism 7.0. The statistical difference in ELISAs and microneutralization assays was determined using one-way analysis of variance (ANOVA), and corrected for multiple comparisons using Dunnett’s test to compare the mean of each group with the mean of the wild type group, where one independent variable was considered (HA titer in microneutralization (MN) assay; AUC in ELISAs). The statistical analysis was performed using two-way ANOVA corrected for multiple comparisons using Dunnett’s test in the challenge studies to compare the mean of each group to the mean of the cHA Ca2 M2 group, where two independent variables—weight loss and time points—were considered. 

## 3. Results

### 3.1. Construction of an Influenza Virus Expressing the M2e Epitope in the Ca2 Antigenic Site of Hemagglutinin

**Figure 1:**
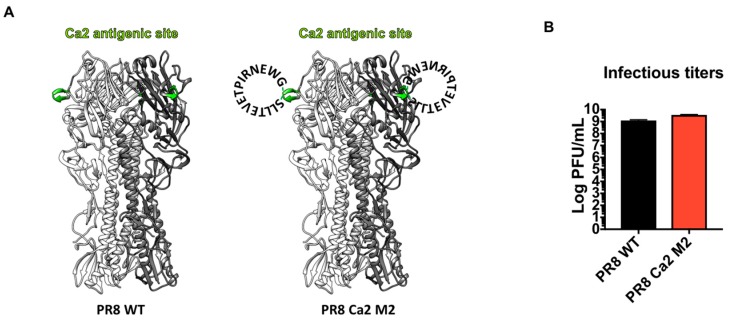
Design and characterization of the PR8 Ca2 M2 virus. (**A**) Illustration of the PR8 HA (left) and PR8 HA displaying the M2e epitope (right). HA trimer based on the PR8 HA (PDB code: 1RU7) is shown. The Ca2 antigenic site is shown in green. The M2e epitope is depicted as a circular amino acid sequences around the Ca2 antigenic site within each monomer (The depiction of the epitope is omitted for the third monomer in this graph). (**B**) Infectious titers of the PR8 WT virus and PR8 Ca2 M2 viral stocks. The PR8 WT (black bar) and PR8 Ca2 M2 (red bar) virus’s stocks were grown in 10-day embryonated chicken eggs. The infectious titers of the viral stocks were determined by plaque assay on Madin–Darby canine kidney (MDCK) cells.

The classical antigenic sites of influenza viruses H1, H3 and B’s HA hemagglutinins have been defined through the observation of natural isolates and the characterization of escape mutants of monoclonal antibodies [70,71,72,73]. The immunodominance of these major antigenic sites has also been investigated [66,74,75,76,77,78], and correlates with the rapid antigenic drift of these sites under immune pressures. To overcome the poor immunogenicity of the M2 protein in the inactivated influenza virus vaccine (IIV) platform, we aimed to improve the antigenic visibility of the M2e by grafting the consensus epitope into one of the major antigenic sites of H1 hemagglutinin (Sa, Sb, Ca1, Ca2 or Cb). We inserted, into the Ca2 antigenic site of PR8 HA, a 15-amino acid epitope (aa ^2^SLLTEVETPIRNEWG^16^) of the human consensus sequence, which was generated by alignment of M2e sequences of human influenza A viruses from previous studies [30,68,69] (Figure 1A). This virus was rescued in the PR8 backbone and designated PR8 Ca2 M2. The presence of the M2e epitope was confirmed by Sanger sequencing. As the major antigenic sites of the HA are often hypervariable and more tolerant to insertions [64,79], we expected the modified HA to still be functional. Indeed, the PR8 Ca2 M2 virus grew to a high titer in embryonated chicken eggs, comparable to the wild type (WT) PR8 virus (Figure 1B). 

### 3.2. M2e Epitope Displayed by the Hemagglutinin Induced Strong M2e-Specific Antibody Responses

**Figure 2:**
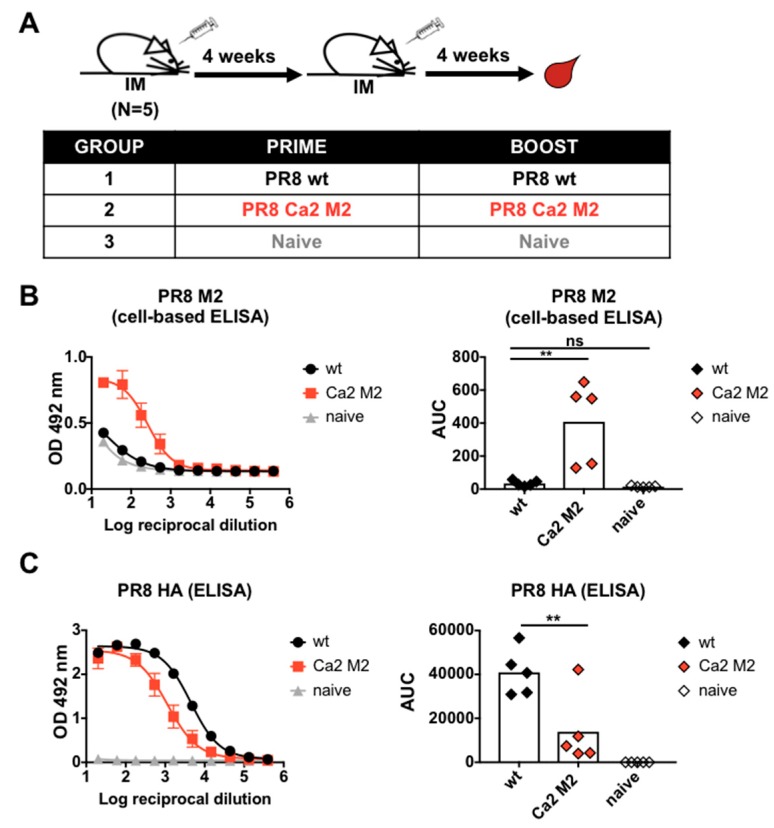
Immunogenicity of the M2e epitope in mice. (**A**) Immunization regimen and groups. Mice were given 10 µg of inactivated purified virus intramuscularly per mouse using a prime-boost vaccination regimen in 4-week intervals. Three groups were included, the PR8 WT group, the PR8 Ca2 M2 group and the naïve group (*n* = 5). Mice were bled 4 weeks after the boost for serological assays and passive serum transfer study. (**B**) Serum M2e-specific IgG responses. Cell-based ELISA using transfected 293T cells was performed to measure M2e-specific serum IgG responses. (**C**) Serum HA-specific IgG responses. ELISA was performed using trimeric recombinant PR8 HA protein as substrate. Area under the curve (AUC) was analyzed as the readout in the ELISA. Statistical difference was determined using one-way ANOVA corrected for multiple comparisons using Dunnett’s test (ns, not significant; *, *p* < 0.05; **, *p* < 0.01; ***, *p* < 0.001; ****, *p* < 0.0001).

To examine the immunogenicity of the PR8 Ca2 M2 virus, we generated inactivated purified virus preparations by inactivating the viruses with 0.03% formaldehyde and purifying them through a 30% sucrose cushion. Viruses were re-suspended in PBS as immunogens. BALB/c mice were immunized with 10 µg of PR8 WT virus or PR8 Ca2 M2 virus per mouse (*n* = 5) with AddaVax as an adjuvant. Mice were primed and boosted 4 weeks later with the same virus. A naïve group was used as a negative control. Mice were bled 4 weeks post-boost to examine serum antibody responses (Figure 2A). Serum IgG titers against the M2e were measured by cell-based ELISA using 293T cells transiently expressing the membrane-bound PR8 M2 protein. Vaccination with PR8 Ca2 M2 virus induced strong M2e-specific antibody responses compared to vaccination with the WT virus (Figure 2B). To determine if disruption of the Ca2 major antigenic site with M2e epitope interferes with antibody responses against PR8 HA, serum IgG titers against recombinant PR8 HA protein were measured by ELISA. As expected, vaccination with the M2e insertion mutant resulted in reduction of HA-specific antibody responses compared to vaccination with wild-type virus (Figure 2C). 

### 3.3. The M2e-Specific Antibody Responses are Non-Neutralizing, ADCC-Active, and Confer Protection in Mice 

**Figure 3:**
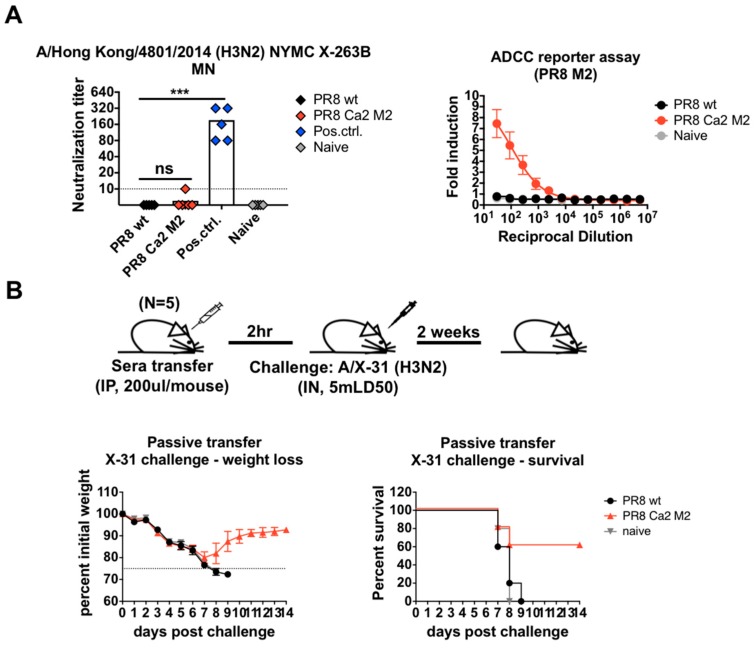
Activities and protectiveness of M2e-specific antibodies induced by the PR8 Ca2 M2 virus. (**A**) M2e-specific antibodies are non-neutralizing but engage in Fc-mediated effector functions. Microneutralization (MN) assay was performed using a recombinant H3N2 virus expressing the heterosubtypic HA and NA in the PR8 backbone. Mouse sera generated by sublethal infection with the same H3N2 virus was used as positive controls. HA assay was used as the readout for the MN assay. ADCC reporter assay was performed using transfected 293T cells transiently expressing the PR8 M2. Fold induction of the reporter signals from the sera over those from the blank were analyzed. Statistical difference was determined using one-way ANOVA corrected for multiple comparisons using Dunnett’s test (ns, not significant; *, *p* < 0.05; **, *p* < 0.01; ***, *p* < 0.001; ****, *p* < 0.0001). (**B**) Passive serum transfer and virus challenge study. Two hundred microliters of pooled sera from each group were transferred into naïve mice intraperitoneally (IP). Mice were challenged with the X-31 virus at a dose of 5x LD_50_ two hours after serum transfer. Weight loss and survival were monitored for two weeks.

Previous reports indicate that anti-M2e antibodies are largely non-neutralizing, and confer protection through the constant region of the antibody (Fc)-mediated effector functions, such as antibody-dependent cell-mediated phagocytosis (ADCP) by macrophages, antibody-dependent cell-mediated cytotoxicity (ADCC) by natural killer (NK) cells or complement-dependent cytotoxicity (CDC) [6,80]. To assess the neutralizing capabilities of induced M2e-specific antibody responses, we performed a microneutralization (MN) assay against a reassortant H3N2 virus with matched M2 protein, in which the heterosubtypic HA and NA are from A/Hong Kong/4801/2014 H3N2, while the other 6 segments are from PR8 (Table 1). Mouse antisera raised against this virus by sub-lethal infection were used as positive controls. Both the PR8 WT sera and PR8 Ca2 M2 sera showed little to no neutralizing activity (Figure 3A). To assess if M2e antibodies engaged in Fc-mediated effector functions, an ADCC reporter assay was performed using 293T cells transiently expressing the PR8 M2 protein. In contrast to naïve and PR8 WT sera, only the PR8 Ca2 M2 sera showed induction of the reporter signal, suggesting that M2e-specific antibody responses have ADCC activity (Figure 3A). 

To evaluate the protectiveness of the M2e antibodies elicited using this approach, a passive serum transfer and virus challenge study was performed in mice. The reassorted X-31 (H3N2) virus (Table 1) was used for challenge to exclude HA and NA-based protection. Pooled sera from each group were transferred into naïve BALB/c mice intraperitoneally (IP). Two hours after the transfer, mice were challenged with 5x LD_50_ of X-31 virus. Weight loss and survival were monitored for two weeks (Figure 3B). PR8 Ca2 M2 sera reduced mortality by 60% compared to PR8 WT sera. All mice in the PR8 WT and naïve groups succumbed to infection with similar progression of morbidity, suggesting that PR8 WT sera offered no protective benefit in the context of challenge study using virus expressing the heterosubtypic HA and NA (Figure 3B). These results suggest that vaccination with novel PR8 Ca2 M2 constructs induces effective M2e based protection, and that this protection is largely mediated by Fc-mediated effector functions. 

### 3.4. Sequential Immunization with Chimeric Hemagglutinins Displaying the Same M2e Epitope Significantly Increased M2e-Specific Antibody Responses and Boosted HA Stalk Antibody Responses

**Figure 4:**
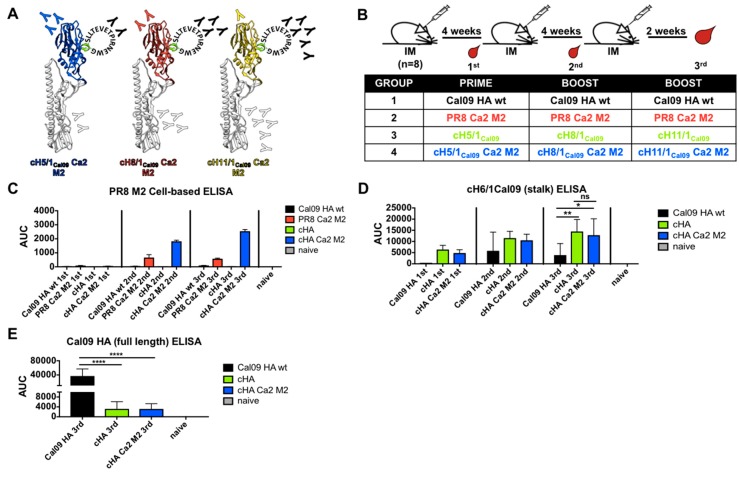
Antibody responses induced by sequential vaccination of cHA Ca2 M2 viruses. (**A**) Illustration of the cHA Ca2 M2 hemagglutinins. The same M2e epitope was inserted into the putative Ca2 antigenic site of cH5/1_Cal09_, cH8/1_Cal09_ and cH11/1_Cal09_ (blue: H5 head; red: H8 head; yellow: H11 head; white: Cal09 HA stalk; green: putative Ca2 antigenic site; black: the M2e epitope; “Y”: expected antibody responses). (**B**) Vaccination regimen and groups. Mice were given 10 µg inactivated purified virus intramuscularly per mouse using a prime-boost-boost vaccination regimen in 4-week intervals. Mice were bled pre-boosts and 2 weeks after the last boost, designated 1st, 2nd and 3rd immunization respectively. Four groups of mice were included (*n* = 8), the WT Cal09 HA group, the PR8 Ca2 M2 group, the cHA group and the cHA Ca2 M2 group. (**C**) Cell-based ELISAs using MDCK cells stably expressing the PR8 M2 protein were performed to measure the progression of M2e-specific antibody responses induced by each immunization (pooled sera). (D and E) ELISAs using recombinant proteins were performed to measure (**D**) the progression of stalk-specific antibody responses and (**E**) total antibody responses to the full length Cal09 HA after the third immunization. Area under the curve (AUC) was used as the readout of the ELISAs. Statistical difference was determined using one-way ANOVA corrected for multiple comparisons using Dunnett’s test (ns, not significant; *, *p* < 0.05; **, *p* < 0.01; ***, *p* < 0.001; ****, *p* < 0.0001).

Previous work from our lab has demonstrated that sequential immunization of mice and ferrets with viruses containing chimeric HAs that share the same stalk sequence but different avian head domains re-directs the immune system to target the conserved, immunosubdominant stalk domain [54,55,56,57,58,59,60,61,62]. We aimed to combine this approach with M2e epitope insertion to boost both M2 and HA stalk antibodies by sequential immunization. The 15-amino acid M2e epitope described above was inserted into the putative Ca2 antigenic sites of cH5/1_Cal09_, cH8/1_Cal09_ and cH11/1_Cal09_ HAs. All the cHAs share the stalk domain from A/California/04/2009 (Cal09) HA. Modified cHA recombinant viruses were rescued in the PR8 backbone and designated cH5/1_Cal09_ Ca2 M2, cH8/1_Cal09_ Ca2 M2, and cH11/1_Cal09_ Ca2 M2 (Figure 4A). We hypothesized that sequential immunization with cHA Ca2 M2 viruses would focus the immune system against the repeatedly exposed M2e epitope as well as the Cal09 stalk epitopes. However, due to the immunodominance of the Ca2 antigenic site relative to the stalk domain, a reduction of stalk antibody titer would be expected. To examine antibody responses in mice, we employed a prime-boost-boost vaccination regimen in 4-week intervals for 4 groups (*n* = 8) (Figure 4B). To compare stalk antibody responses, one group received corresponding cHA viruses without the Ca2 M2 epitopes. To determine if sequential immunization of cHA Ca2 M2 constructs is superior, one group was immunized repeatedly with PR8 Ca2 M2 virus. As a wild type HA control, one group received repeated immunization with virus expressing Cal09 HA in a PR8 backbone. Mice were bled pre-boosts and terminally bled 2 weeks after the last boost to examine the progression of antibody responses (Figure 4B). 

To conveniently measure M2e-specific antibody titers, a PR8 M2-expressing MDCK cell line was generated for use in cell-based ELISAs. Pooled sera from each group were used to evaluate the progression of M2e antibody responses. Three technical replicates were performed for each group at each time point. Pooled naïve sera were used as negative controls. After the first boost (2nd immunization), both the PR8 Ca2 M2 group and the cHA Ca2 M2 group showed increased M2e antibody responses, with the cHA Ca2 M2 titer being about 2.8-fold higher than the PR8 Ca2 M2 titer (Figure 4C). The second boost (3rd immunization) of cHA Ca2 M2 virus further increased the M2e antibody responses, with about a 4.4-fold improvement over the PR8 Ca2 M2 response (Figure 4C). Interestingly, the second boost of the PR8 Ca2 M2 virus did not improve M2e antibody titers, which could be attributed to repeated exposure of other immunodominant antigenic sites on the PR8 HA. These data confirmed that sequential immunization with cHA Ca2 M2 viruses induced substantially higher M2e antibody responses than repeated immunizations with PR8 Ca2 M2 virus, and that immunization with WT Cal09 HA and unmodified cHAs induced negligible M2e antibody responses. To determine if the stalk antibody responses are boosted in the cHA Ca2 M2 group, anti-HA stalk titers were assessed by ELISA against recombinant cH6/1_Cal09_ protein. cHA Ca2 M2 viruses induced stalk antibodies at a similar level to unmodified cHA viruses (Figure 4D). As expected, both the cHA and cHA Ca2 M2 groups induced significantly higher stalk antibody responses than the group that was repeatedly immunized with the WT Cal09 HA virus (Figure 4D), which showed the highest total antibody responses against the full length Cal09 HA (Figure 4E). 

### 3.5. Sequential Immunization with cHA Ca2 M2 Viruses Conferred Antibody-Mediated Protection against Viruses Expressing Heterosubtypic or Homologous HA and NA in Mice

**Figure 5:**
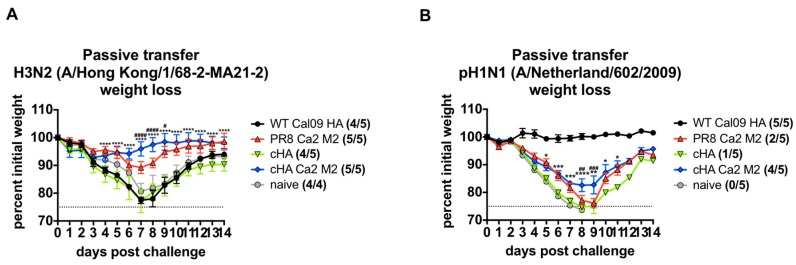
Sequential immunizations with cHA Ca2 M2 viruses elicits protective antibody responses against both viruses expressing heterosubtypic or homologous HA and NA in mice. (**A**) cHA Ca2 M2 immunizations elicited more protective antibody responses against the A/Hong Kong/1/68-2-MA21-2 H3N2 challenge compared to the PR8 Ca2 M2 immunization. (**B**) cHA Ca2 M2 immunizations elicited more protective antibody responses against A/Netherland/602/2009 pdm in the H1N1 challenge compared to both PR8 Ca2 M2 and cHA immunizations. Weight loss and survival (survived/total) were monitored for two weeks. The statistical difference of the weight loss was determined using two-way ANOVA corrected for multiple comparisons using Dunnett’s test. Statistical significances between cHA Ca2 M2 group versus cHA group and cHA Ca2 M2 group versus PR8 Ca2 M2 group are shown in “*” and “^#^” respectively; (*, and ^#^, *p* < 0.05; **, and ^##^, *p* < 0.01; ***, and ^###^, *p* < 0.001; ****, and ^####^, *p* < 0.0001).

Passive serum transfer and virus challenge studies were performed in mice to evaluate the protectiveness of M2 antibodies induced by the cHA Ca2 M2 viruses. We first examined if the M2e antibody responses could protect against an H3N2 virus. Here, we chose the mouse-adapted A/Hong Kong/1/68-2-MA21-2 H3N2 strain (Table 1) for challenge as it was a more genuine strain than the reassorted lab virus X-31. Equal amounts of sera from terminally bled mice within each group were pooled. We transferred 200 µL of pooled sera to each mouse intraperitoneally (IP). Two hours after serum transfer, mice were infected with the A/Hong Kong/1/68-2-MA21-2 H3N2 virus at a dose of 5x LD_50_. Weight loss and survival were monitored for two weeks. Since the virus dose at 5x LD_50_ was not completely lethal, causing little or no mortality in the negative control groups (naïve, Cal09 HA and cHA sera), differences in morbidity as determined by weight loss for each group were compared. The protective efficacy of cHA Ca2 M2 sera was significantly better than that of PR8 Ca2 M2 sera, with mice experiencing minimal weight loss (Figure 5A). As expected, heterosubtypic H1N1 sera from the WT Cal09 HA and cHA group failed to prevent weight loss, resulting in morbidity comparable to mice receiving naïve sera (Figure 5A). These results suggest that higher M2e antibody responses elicited by the cHA Ca2 M2 immunization provided better protection against a virus with heterosubtypic HA and NA in mice. 

To differentiate the protectiveness of different sera against a homologous H1N1 virus challenge, we transferred 100 µL of pooled sera into naïve mice intraperitoneally (IP). Two hours later, mice were challenged with 5x LD_50_ of A/Netherland/602/2009 pdm H1N1 (Table 1). Weight loss and survival were monitored over 2 weeks. Homologous WT Cal09 HA sera were used as the positive controls, providing full protection and preventing weight loss. Since the sera contain matched-stalk but mismatched-NA (PR8 NA) antibodies, and they were passively transferred in a suboptimal amount (100 µL). High morbidity and mortality (1/5) was observed in the cHA group compared to our previous studies [55,61]. The PR8 Ca2 M2 sera provided slightly better protection, but also resulted in high mortality (2/5). Importantly, cHA Ca2 M2 sera conferred significantly better protection than both PR8 Ca2 M2 sera and cHA sera, likely due to the combination of boosted M2 and H1 stalk antibodies (Figure 5B). Thus, in the context of challenges with viruses expressing heterosubtypic HA and NA or homologous HA, the group immunized with cHA Ca2 M2 sera conferred superior antibody-mediated protection compared to strategies that relied solely on M2 or HA stalk.

## 4. Discussion

Vaccination platforms targeting the stalk domain of the hemagglutinin (HA) or the extracellular domain of the matrix protein 2 (M2e) have been extensively developed as potential universal vaccine candidates due to the conserved nature of these regions. Here, we report a novel inactivated influenza virus vaccine approach that combines these two strategies by inserting a consensus M2e epitope into the immunodominant antigenic site of chimeric HAs (cHA). This modification successfully enhances the immunogenicity of the M2e epitope by improving its antigenic visibility without compromising the integrity of the anti-stalk immune response. Since M2-based vaccine candidates predominantly confer antibody-mediated protection [6], passive serum transfer/viral challenge instead of direct viral challenge after immunization was performed to exclude possible interference from cell-mediated immunity, which could also be elicited by inactivated viruses. Serum antibodies induced by immunization with the cHA Ca2 M2 constructs provided superior protection in both challenge studies against viruses expressing heterosubtypic or homologous HA and NA, in which M2e antibodies elicited by the inserted epitope target both matched (H3N2) and mismatched (pdm H1N1) M2 proteins. Therefore, although we have only tested a historical A/Hong Kong/1968 H3N2 virus in our challenge study, due to the limited selection of virulent H3N2 strains in mice, we would expect a similar outcome if we were to test a contemporary H3N2 strain containing a mismatched M2 protein. The protective efficacy of elicited M2e antibodies is most likely the result of Fc-mediated effector functions. The advantage of this vaccine approach is that it can easily be applied to inactivated or live-attenuated influenza virus vaccine (LAIV) platforms for both H1N1 and H3N2 viruses. Furthermore, these vaccine candidates could be potentially used for pandemic preparedness, since they elicit antibody responses that focus on the conserved stalk and M2e.

To optimize this approach in the future, a variety of human or avian M2e consensus epitopes could be inserted into different classical antigenic site(s) of the HA instead of, or in addition to, the Ca2 site, such as the Sa and Sb sites, which have been suggested to be more immunodominant than the Ca2 site in animal models [74,75]. Insertion of the M2e epitope into different antigenic sites might change the induction of M2e and stalk antibodies due to variations in immunodominance that are not yet fully understood. Therefore, it would be important to test a panel of designs in vivo to determine overall protectiveness. Importantly, it would also be feasible to construct similar group 2 cHA viruses containing the consensus M2e epitopes. Formulation of a bi-valent vaccine containing both groups of modified cHAs could be tested to evaluate breadth of cross protection. Since the cHAs could be engineered to contain exotic avian hemagglutinin head domains, they could also potentially contribute to protection of pre-pandemic avian influenza viruses, such as H5N1 or H7N9 viruses, although that was not evaluated in the present study.

In our study, two doses of cHA Ca2 M2 viruses were sufficient to elicit superior M2e antibody responses in mice, while a third immunization further increased said antibody titer. It has been shown that humans naturally infected with pandemic H1N1 virus, which is antigenically distinct from previous seasonal H1N1 viruses, have detectable serum M2 antibody responses. This suggests that humans have pre-existing immunity against the M2 protein [10]. Our previous study showed that H1 (group 1 HA) stalk antibodies could be effectively boosted by an H5N1 (group 1 HA) vaccine in humans, as most humans were primed against the H1 stalk, which is similar to the H5 stalk [81]. Therefore, we hope that just two doses of sequential immunization or even one immunization using cHA Ca2 M2 viruses might be sufficient to mount long-lasting protective M2 antibody responses in humans who are primed against the M2e. This would replace annual re-vaccinations. Our approach allows for the construction of LAIV expressing cHA Ca2 M2 hemagglutinins to investigate whether this strategy can potentially confer better mucosal and cellular immunity, in addition to the humoral immunity against M2e [82,83,84,85].

## Figures and Tables

**Table 1:** The M2e sequences of test viruses.

Name	M2e Sequences (Amino Acid 1-24)
Human consensus [30,68,69]	MSLLTEVETPIRNEWGCRCNDSSD
* A/Hong Kong/4801/2014 H3N2 NYMC X-263B	MSLLTEVETPIRNEWGCRCN**G**SSD
* A/Hong Kong/1/1968 H3N2 X-31	MSLLTEVETPIRNEWGCRCN**G**SSD
A/Netherland/602/2009 H1N1	MSLLTEVETP**T**R**S**EW**E**CRC**S**DSSD
A/Hong Kong/1/1968-2-MA21-2 H3N2	MSLLTEVETPIRNEWGCRCNDSSD

Amino acids that are different from human consensus are in bold and underlined; * Reassortant viruses that have six genomic segments from PR8 except the HA and NA. The M2 proteins of these viruses are from PR8.
